# Crystal structure and Hirshfeld surface analysis of 2-[(1,3-benzoxazol-2-yl)sulfan­yl]-*N*-(2-meth­oxy­phen­yl)acetamide

**DOI:** 10.1107/S2056989019012908

**Published:** 2019-09-27

**Authors:** Abdullah Aydin, Sevim Turktekin Celikesir, Mehmet Akkurt, Merve Saylam, Varol Pabuccuoglu

**Affiliations:** a Department of Mathematics and Science Education, Faculty of Education, Kastamonu University, 37200 Kastamonu, Turkey; b Department of Physics, Faculty of Sciences, Erciyes University, 38039 Kayseri, Turkey; c Department of Pharmaceutical Chemistry, Faculty of Pharmacy, Izmir Katip Celebi University, 35620 Izmir, Turkey; d Department of Pharmaceutical Chemistry, Faculty of Pharmacy, Ege University, 35100 Izmir, Turkey

**Keywords:** crystal structure, 1,3-benzoxazole ring system, dimers, hydrogen bonding, Hirshfeld surface analysis

## Abstract

In the title compound, C_16_H_14_N_2_O_3_S, the 1,3-benzoxazole ring system is essentially planar (r.m.s deviation = 0.004 Å) and makes a dihedral angle of 66.16 (17)° with the benzene ring of the meth­oxy­phenyl group. Two intra­molecular N—H⋯O and N—H⋯N hydrogen bonds occur, forming *S*(5) and *S*(7) ring motifs, respectively. In the crystal, pairs of C—H⋯O hydrogen bonds link the mol­ecules into inversion dimers with *R* ^2^_2_(14) ring motifs, stacked along the *b*-axis direction. The inversion dimers are linked by C—H⋯π and π–π-stacking inter­actions [centroid-to-centroid distances = 3.631 (2) and 3.631 (2) Å], forming a three-dimensional network. Two-dimensional fingerprint plots associated with the Hirshfeld surface show that the largest contributions to the crystal packing come from H⋯H (39.3%), C⋯H/H⋯C (18.0%), O⋯H/H⋯O (15.6) and S⋯H/H⋯S (10.2%) inter­actions.

## Abstract 2

In the title compound, there are two intra­molecular N—H⋯O and N—H⋯N hydrogen bonds, forming *S*(5) and *S*(7) ring motifs, respectively. In the crystal, pairs of C—H⋯O hydrogen bonds link mol­ecules into inversion dimers with 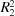
(14) ring motifs, stacked along the *b-*axis direction. The inversion dimers are linked by C—H⋯π and π–π-stacking inter­actions, forming a three-dimensional network.

## Chemical context   

As a part of our ongoing research on synthesis and screening of pharmacological activities of compounds with a benzoxazole ring, which is known to produce a wide range of biological activities (Aggarwal *et al.*, 2017 ▸; Gautam *et al.*, 2012 ▸), we have focused on the synthesis of 3-substituted benzoxazolone-2-thione and S-substituted benzoxazole-2-thiol derivatives. It is well known that alkyl­ation of benzoxazolone-2-thione leads to the *S*-alkyl­ated derivatives instead of *N*-alkyl­ated ones (Xiang *et al.*, 2012 ▸; Rakse *et al.*, 2013 ▸; Yurttaş *et al.*, 2015 ▸). In this manner, the title compound was synthesized as a member of the target S-substituted benzoxazole-2-thiol series. The title compound is listed in the literature with registry number CASRN 331966-95-1 but corresponding scientific reference data are not available.
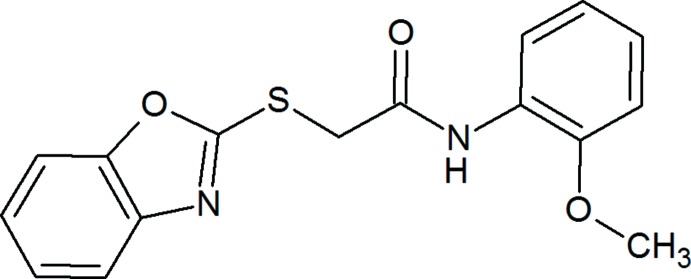


## Structural commentary   

**Figure 1:**
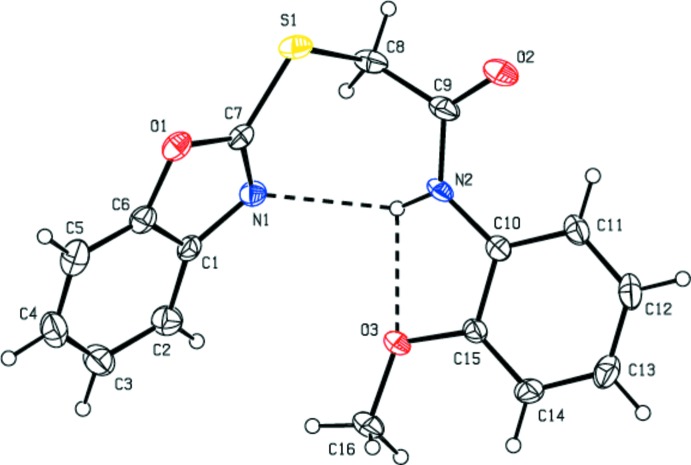
The mol­ecular structure of the title compound, showing the atom labelling and displacement ellipsoids drawn at the 30% probability level. Intra­molecular hydrogen bonds are shown as dashed lines.

In the mol­ecular structure of the title compound (Fig. 1 ▸), the 1,3-benzoxazole ring system (N1/O1/C1–C7) is essentially planar (r.m.s deviation = 0.004 Å) and makes a dihedral angle of 66.16 (17)° with the benzene ring (C10–C15) of the meth­oxy­phenyl group. Atoms O3 and C16 deviate from the benzene ring by −0.008 (3) and 0.099 (6) Å, respectively. The torsion angle C7—S1—C8—C9 = −87.7 (3)°, S1—C8—C9— N2 = 91.6 (4)° and C8—C9—N2—C10 = −178.8 (3)°. The C7—S1 [1.740 (4) Å] and C8—S1 [1.812 (4) Å] bond lengths are comparable with those reported for three similar structures, *viz.* 2-[(4,6-di­amino­pyrimidin-2-yl)sulfan­yl]-*N*-(2-meth­yl­phen­yl)acetamide (1.763 and 1.805 Å, respectively; Subasri *et al.*, 2014 ▸), 2-[(4,6-di­amino­pyrimidin-2-yl)sulfan­yl]-*N*-(2,4-di­methyl­phen­yl)acetamide [1.7650 (14) and 1.8053 (16) Å, respectively; Choudhury *et al.*, 2017 ▸] and 2-[(4,6-di­amino­pyrimidin-2-yl)sulfan­yl]-*N*-(3-meth­oxy­phen­yl) acetamide [1.7721 (17) and 1.8126 (18) Å, respectively; Choudhury *et al.*, 2017 ▸]. The two intra­molecular hydrogen bonds, N2—H*N*2⋯O3 and N2—H*N*2⋯N1, form *S*(5) and *S*(7) ring motifs, respectively (Table 1 ▸, Fig. 1 ▸).

## Supra­molecular features   

**Figure 2:**
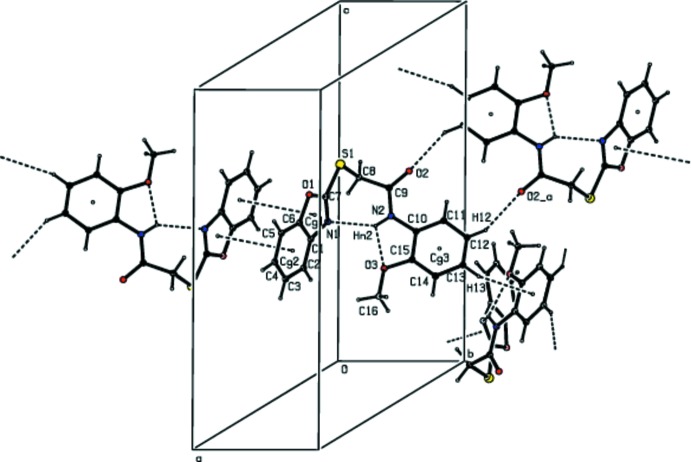
A packing diagram of the title compound, showing the intra- and inter­molecular N—H⋯N and N—H⋯O, C—H⋯O hydrogen bonds, C—H⋯π inter­actions and π–π-stacking inter­actions (dashed lines). Symmetry code: (*a*) − *x*, 2 − *y*, 1 − *z*.

**Figure 3:**
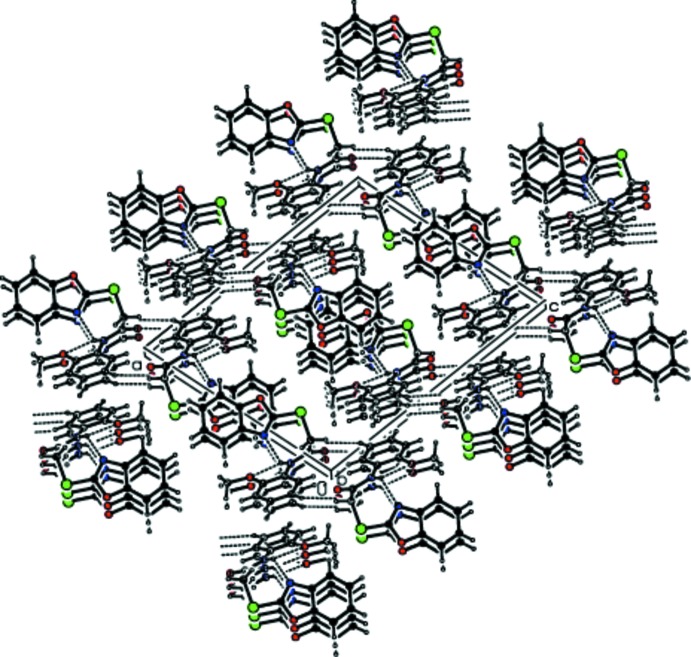
Packing diagram of the title compound viewed down the *b* axis.

In the crystal, pairs of C—H⋯O hydrogen bonds link the mol­ecules into inversion dimers with 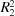
(14) ring motifs, stacking along the *b-*axis direction. These dimers are linked by C—H⋯π (Table 1 ▸, Fig. 2 ▸) and π–π-stacking inter­actions [Fig. 2 ▸; distances of 3.631 (2) and 3.631 (2) Å between the centroids of the five- and opposite six-membered rings of the 1,3-benzoxazole ring system of adjacent mol­ecules], forming a three-dimensional network (Fig. 3 ▸).

## Hirshfeld surface analysis   

**Figure 4:**
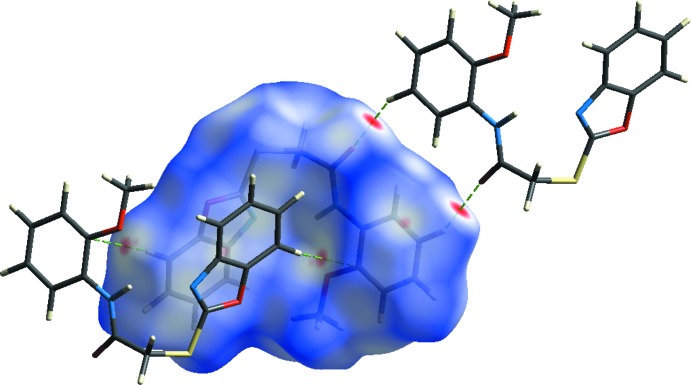
Hirshfeld surface mapped over *d*_norm_, showing the weak inter­molecular C—H⋯O and C—H⋯C contacts.

**Figure 5:**
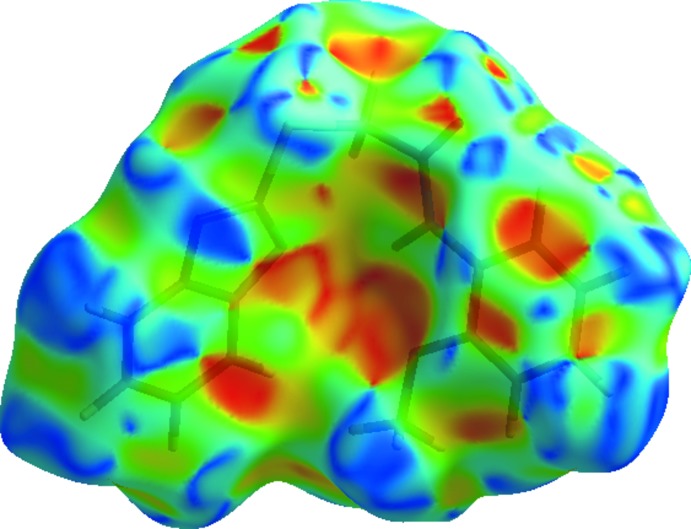
View of the three-dimensional Hirshfeld surface of the title compound plotted over electrostatic potential energy in the range −0.0500 to 0.0500 a.u. using the STO-3 G basis set at the Hartree–Fock level of theory. Hydrogen- bond donors and acceptors are shown as blue and red regions around the atoms corresponding to positive and negative potentials, respectively.

In order to explore the role of weak inter­molecular inter­actions in the crystal packing, Hirshfeld surfaces (*d*_norm_) and the related two-dimensional fingerprint plots were generated using *CrystalExplorer17.5* (Spackman & Jayatilaka, 2009 ▸; Wolff *et al.*, 2012 ▸). The three-dimensional mol­ecular Hirshfeld surfaces were generated using a high standard surface resolution over a colour scale of −0.1599 to 1.2011 a.u. for *d*_norm_ (Fig. 4 ▸). The red spots in the Hirshfeld surface represent short N⋯H/H⋯N and O⋯H/H⋯O contacts. On the shape-index surface (Fig. 5 ▸), convex blue regions represent hydrogen-donor groups and concave red regions represent hydrogen-acceptor groups. In addition, concave red regions represent C—H⋯π and π–π inter­actions.

**Figure 6:**
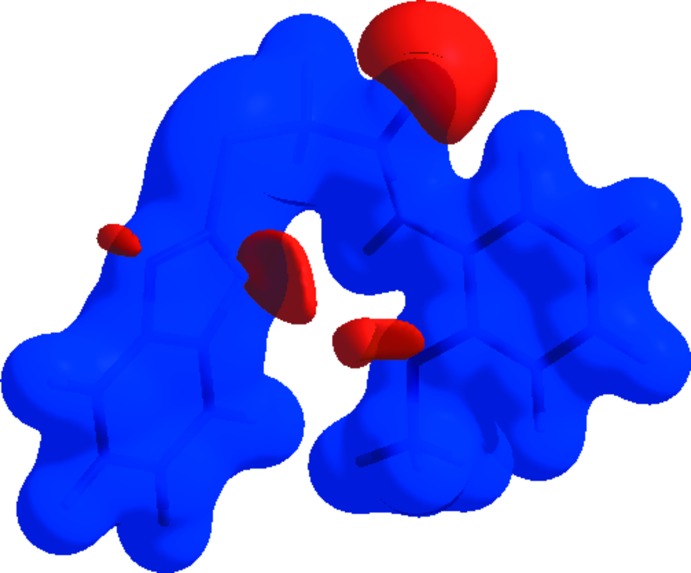
Hirshfeld surfaces for the title compound, mapped with shape-index.

The bright-red spots indicate their roles as the respective donors and/or acceptors; they also appear as blue and red regions corresponding to positive and negative potentials on the Hirshfeld surface mapped over electrostatic potential (Spackman *et al.*, 2008 ▸) shown in Fig. 6 ▸. The blue regions indicate the positive electrostatic potential (hydrogen-bond donors), while the red regions indicate the negative electrostatic potential (hydrogen-bond acceptors).

**Figure 7:**
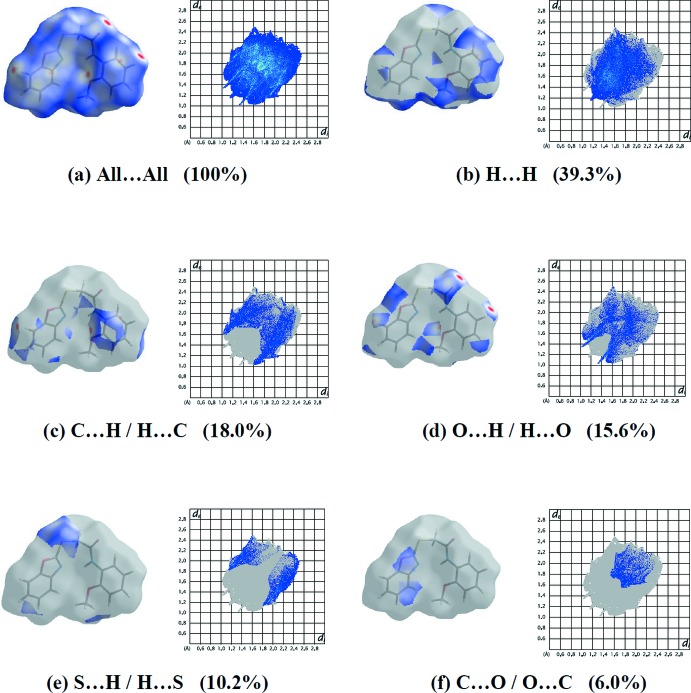
Hirshfeld surfaces and two-dimensional fingerprints for the compound, showing (*a*) all inter­actions and those delineated into (*b*) H⋯H, (*c*) C⋯H/H⋯C, (*d*) O⋯H/H⋯O, (*e*) S⋯H/H⋯S and (*f*) C⋯O/O⋯C contacts.

The two-dimensional fingerprint plots (Fig. 7 ▸) qu­antify the contributions of each type of inter­molecular inter­action to the Hirshfeld surface (McKinnon *et al.*, 2007 ▸). The largest contribution (39.3% of the surface) is from H⋯H contacts (Table 2 ▸), which represent van der Waals inter­actions, followed by C⋯H/H⋯C contacts involved in C—H⋯π inter­actions (18.0%). Finally, the O⋯H/H⋯O (15.6%), S⋯H/H⋯S (10.2%) and C⋯C (4.5%) contacts correspond to hydrogen bonds and π–π inter­actions, respectively. The percentage contributions to the Hirshfeld surface of the various inter­atomic contacts are given in Table 3 ▸.

## Database survey   

Related compounds to the title compound include 2-[(4,6-di­amino­pyrimidin-2-yl)sulfan­yl]-*N*-(naphthalen-1-yl)acetamide (refcode JARPOK; Subasri *et al.*, 2017 ▸), 2-[(4,6-di­amino­pyrimidin-2-yl)sulfan­yl]-*N*-(4-fluoro­phen­yl)acetamide (JAR­PUQ; Subasri *et al.*, 2017 ▸), 2-[(4,6-di­amino­pyrimidin-2-yl)sulf­an­yl]-*N*-(2-methyl­phen­yl)acetamide (GOKWIO; Subasri *et al.*, 2014 ▸), 2-[(4,6-di­amino­pyrimidin-2-yl)sulfan­yl]-*N*-(2,4-di­methyl­phen­yl)acetamide (JAXFIA; Choudhury *et al.*, 2017 ▸), 2-[(4,6-di­amino­pyrimidin-2-yl)sulfan­yl]-*N*-(3-meth­oxy­phen­yl) acetamide (refcode: JAXFOG; Choudhury *et al.*, 2017 ▸) and 2-[(2-amino­phen­yl)sulfan­yl]-*N*-(4-meth­oxy­phen­yl)acetamide (PAXTEP; Murtaza *et al.*, 2012 ▸).

In the crystals of JARPOK and JARPUQ, mol­ecules are linked by pairs of N—H⋯N hydrogen bonds, forming inversion dimers with 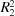
(8) ring motifs. In the crystal of JARPOK, the dimers are linked by bifurcated N—H⋯(O,O) and C—H⋯O hydrogen bonds, forming layers parallel to (100). In the crystal of JARPUQ, the dimers are linked by N—H⋯O hydrogen bonds, also forming layers parallel to (100). The layers are linked by C—H⋯F hydrogen bonds, forming a three-dimensional architecture.

In the crystal of GOKWIO, mol­ecules are linked *via* pairs of N—H⋯N hydrogen bonds, forming inversion dimers with an 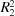
(8) ring motif. The dimers are linked by N—H⋯O and C—H⋯O hydrogen bonds, forming sheets parallel to (100).

In the crystals of JAXFIA and JAXFOG, a pair of N—H⋯N hydrogen bonds links the mol­ecules, forming inversion dimers with 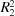
(8) ring motifs. In JAXFIA, the dimers are linked by N—H⋯O and C—H⋯O hydrogen bonds, enclosing 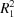
(14), 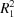
(11) and 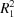
(7) ring motifs, forming layers parallel to the (100) plane. There is also an N—H⋯π inter­action present within the layer. In JAXFOG, the inversion dimers are linked by N—H⋯O hydrogen bonds enclosing an 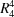
(18) ring motif. The presence of N—H⋯O and C—H⋯O hydrogen bonds generate an 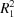
(6) ring motif. The combination of these various hydrogen bonds results in the formation of layers parallel to the (1
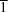
1) plane.

In the crystal of PAXTEP, mol­ecules are consolidated in the form of polymeric chains along [010] as a result of N—H⋯O hydrogen bonds, which generate 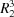
(18) and 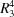
(22) loops. The polymeric chains are inter­linked through C—H⋯O inter­action and complete 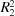
(8) ring motifs.

## Synthesis and crystallization   

The starting materials, 2-mercaptobenzoxazole and α-chloro-*N*-(*o*-meth­oxy­phen­yl)acetamide, were synthesized according to literature methods (Maske *et al.*, 2012 ▸; Ren *et al.*, 2015 ▸). For the synthesis of the title compound, 2-mercaptobenzoxazole (1 eq) and α-chloro-*N*-(*o*-meth­oxy­phen­yl) acetamide (1 eq) were heated in acetone under reflux for 1.5 h in the presence of K_2_CO_3_ (1 eq). The reaction mixture was then cooled to room temperature and cold water was added until precipitation was complete. The precipitate was filtered, washed with cold water and dried. The crude product was crystallized from methanol (yield 31%); m.p. 370 K.

^1^H NMR (DMSO-*d*_6_, 400 MHz) δ 3.82 (3H, *s*, OCH_3_), δ 4.42 (2H, *s*, CH_2_), δ 6.89 (1H, *m*, Ar-H), δ 7.03–7.10 (2H, *m*, Ar-H), δ 7.31–7.38 (2H, *m*, Ar-H), δ 7.62–7.68 (2H, *m*, Ar-H), δ 7.97 (1H, *d*, *J* = 8.4 Hz, Ar-H), δ 9.65 (1H, *s*, NH) p.p.m. IR *v*_max_ cm^−1^: 3295 (NH), 1675 (amide I), 1534 (amide II); MS (ESI) *m*/*z* (intensity %): 315.32 (26) [*M*+H]^+^ 192.27 (100).

## Refinement   

Crystal data, data collection and structure refinement details are summarized in Table 4 ▸. All H atoms were positioned with idealized geometry and refined as riding: N—H = 0.86 Å, C—H = 0.93–0.97 Å with *U*_iso_(H) = 1.5*U*_eq_(C) for methyl H atoms and *U*_iso_(H) = 1.2*U*_eq_(C, N) for all other H atoms. Thirty one outliers (13 1 3), (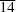 1 1), (8 3 12), (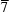 5 15), (0 3 18), (5 0 14), (14 2 5), (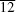 0 14), (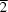 4 17), (14 3 1), (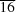 3 5), (1 8 4), (1 4 15), (10 5 2), (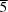 7 8), (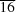 0 10), (14 2 1), (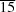 1 1), (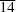 3 12), (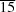 2 7), (4 1 17), (11 0 10), (15 1 2), (3 4 14), (10 2 6), (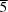 0 18), (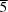 3 18), (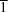 6 11), (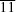 5 2), (10 1 9), (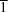
4 1 2) were omitted in the final cycles of refinement.

## Supplementary Material

Crystal structure: contains datablock(s) I. DOI: 10.1107/S2056989019012908/rz5264sup1.cif

Structure factors: contains datablock(s) I. DOI: 10.1107/S2056989019012908/rz5264Isup2.hkl

CCDC reference: 1954699

Additional supporting information:  crystallographic information; 3D view; checkCIF report

## Figures and Tables

**Table 1:** Hydrogen-bond geometry (Å, °) *Cg*3 is the centroid of the C10–C15 benzene ring of the meth­oxy phenyl group.

*D*—H⋯*A*	*D*—H	H⋯*A*	*D*⋯*A*	*D*—H⋯*A*
N2—H*N*2⋯O3	0.86	2.22	2.608 (4)	107
N2—H*N*2⋯N1	0.86	2.39	3.075 (4)	136
C8—H8*A*⋯N1	0.97	2.48	2.914 (5)	107
C11—H11⋯O2	0.93	2.28	2.869 (5)	121
C12—H12⋯O2^i^	0.93	2.52	3.378 (6)	153
C13—H13⋯*Cg*3^ii^	0.93	2.89	3.634 (5)	138

Symmetry codes: (i) 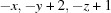; (ii) 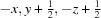.

**Table 2:** Summary of selected short inter­atomic contacts (Å) in the title compound

Contact	Distance	Symmetry operation
H5⋯O3	2.72	1 − *x*, 1 − *y*, 1 − *z*
S1⋯H2	3.10	*x*, 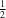 − *y*, 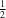 + *z*
C5⋯C1	3.38	1 − *x*, −*y*, 1 − *z*
H8*B*⋯C11	3.06	−*x*, 1 − *y*, 1 − *z*
H12⋯O2	2.52	−*x*, 2 − *y*, 1 − *z*
O2⋯H16*A*	2.74	*x*, 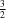 − *y*, 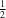 + *z*
C10⋯H13	3.04	−*x*, − 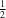 + *y*, 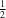 − *z*
C12⋯H8*A*	2.82	*x*, 1 + *y*, *z*

**Table 3:** Percentage contributions of inter­atomic contacts to the Hirshfeld surface of the title compound

Contact	Percentage contribution
H⋯H	39.3
H⋯C/C⋯H	18.0
O⋯H/H⋯O	15.6
S⋯H/H⋯S	10.2
C⋯O/O⋯C	6.0
C⋯C	4.5
N⋯H/H⋯N	4.1
C⋯N/N⋯C	1.4
C⋯S/S⋯C	0.6
N⋯O/O⋯N	0.1

**Table 4:** Experimental details

Crystal data	
Chemical formula	C_16_H_14_N_2_O_3_S
*M*_r_	314.35
Crystal system, space group	Monoclinic, *P*2_1_/*c*
Temperature (K)	296
*a*, *b*, *c* (Å)	13.6670 (13), 6.8704 (6), 16.7220 (16)
β (°)	108.020 (4)
*V* (Å^3^)	1493.1 (2)
*Z*	4
Radiation type	Mo *K*α
μ (mm^−1^)	0.23
Crystal size (mm)	0.10 × 0.07 × 0.06

Data collection	
Diffractometer	Bruker APEXII CCD
Absorption correction	Multi-scan (*SADABS*; Bruker, 2007 ▸)
*T*_min_, *T*_max_	0.654, 0.745
No. of measured, independent and observed [*I* > 2σ(*I*)] reflections	23464, 3019, 2241
*R*_int_	0.092
(sin θ/λ)_max_ (Å^−1^)	0.627

Refinement	
*R*[*F* ^2^ > 2σ(*F* ^2^)], *wR*(*F* ^2^), *S*	0.092, 0.169, 1.10
No. of reflections	3019
No. of parameters	200
H-atom treatment	H-atom parameters constrained
Δρ_max_, Δρ_min_ (e Å^−3^)	0.25, −0.31

Computer programs: *APEX2* and *SAINT* (Bruker, 2007 ▸), *SHELXT2014/5* (Sheldrick, 2015*a* ▸), *SHELXL2018/1* (Sheldrick, 2015*b* ▸), *ORTEP-3 for Windows* and *WinGX* (Farrugia, 2012 ▸) and *PLATON* (Spek, 2009 ▸).

## supplementary crystallographic information

### Crystal data

**Table:**

C_16_H_14_N_2_O_3_S	*F*(000) = 656
*M**_r_* = 314.35	*D*_x_ = 1.398 Mg m^−^^3^
Monoclinic, *P*2_1_/*c*	Mo *K*α radiation, λ = 0.71073 Å
*a* = 13.6670 (13) Å	Cell parameters from 6692 reflections
*b* = 6.8704 (6) Å	θ = 3.2–26.3°
*c* = 16.7220 (16) Å	µ = 0.23 mm^−^^1^
β = 108.020 (4)°	*T* = 296 K
*V* = 1493.1 (2) Å^3^	Block, colourless
*Z* = 4	0.10 × 0.07 × 0.06 mm

### Data collection

**Table:**

Bruker APEXII CCD diffractometer	2241 reflections with *I* > 2σ(*I*)
φ and ω scans	*R*_int_ = 0.092
Absorption correction: multi-scan (SADABS; Bruker, 2007)	θ_max_ = 26.5°, θ_min_ = 3.1°
*T*_min_ = 0.654, *T*_max_ = 0.745	*h* = −17→17
23464 measured reflections	*k* = −8→8
3019 independent reflections	*l* = −20→20

### Refinement

**Table:**

Refinement on *F*^2^	0 restraints
Least-squares matrix: full	Hydrogen site location: inferred from neighbouring sites
*R*[*F*^2^ > 2σ(*F*^2^)] = 0.092	H-atom parameters constrained
*wR*(*F*^2^) = 0.169	*w* = 1/[σ^2^(*F*_o_^2^) + (0.0406*P*)^2^ + 3.9251*P*] where *P* = (*F*_o_^2^ + 2*F*_c_^2^)/3
*S* = 1.10	(Δ/σ)_max_ < 0.001
3019 reflections	Δρ_max_ = 0.25 e Å^−^^3^
200 parameters	Δρ_min_ = −0.31 e Å^−^^3^

### Special details

**Table:**

Geometry. All esds (except the esd in the dihedral angle between two l.s. planes) are estimated using the full covariance matrix. The cell esds are taken into account individually in the estimation of esds in distances, angles and torsion angles; correlations between esds in cell parameters are only used when they are defined by crystal symmetry. An approximate (isotropic) treatment of cell esds is used for estimating esds involving l.s. planes.

### Fractional atomic coordinates and isotropic or equivalent isotropic displacement parameters (Å^2^)

**Table:**

	*x*	*y*	*z*	*U*_iso_*/*U*_eq_	
C1	0.4055 (3)	0.2452 (5)	0.4388 (2)	0.0301 (9)	
C2	0.4121 (3)	0.1973 (6)	0.3599 (3)	0.0437 (11)	
H2	0.353578	0.186794	0.313350	0.052*	
C3	0.5089 (4)	0.1660 (7)	0.3535 (3)	0.0507 (12)	
H3	0.515664	0.134545	0.301349	0.061*	
C4	0.5962 (4)	0.1801 (6)	0.4223 (3)	0.0515 (13)	
H4	0.660182	0.158127	0.415281	0.062*	
C5	0.5910 (3)	0.2258 (6)	0.5012 (3)	0.0460 (11)	
H5	0.649354	0.234724	0.547961	0.055*	
C6	0.4940 (3)	0.2573 (5)	0.5059 (3)	0.0330 (9)	
C7	0.3608 (3)	0.3093 (5)	0.5452 (2)	0.0304 (9)	
C8	0.1670 (3)	0.3513 (7)	0.5515 (3)	0.0401 (10)	
H8A	0.160668	0.258715	0.506432	0.048*	
H8B	0.122257	0.308919	0.583244	0.048*	
C9	0.1330 (3)	0.5503 (6)	0.5143 (2)	0.0368 (10)	
C10	0.1249 (3)	0.7568 (6)	0.3915 (2)	0.0272 (8)	
C11	0.0683 (3)	0.9111 (6)	0.4077 (2)	0.0364 (10)	
H11	0.044048	0.906129	0.453818	0.044*	
C12	0.0479 (3)	1.0720 (7)	0.3557 (3)	0.0438 (11)	
H12	0.008978	1.173897	0.366520	0.053*	
C13	0.0844 (3)	1.0826 (7)	0.2882 (3)	0.0443 (11)	
H13	0.070946	1.191980	0.253773	0.053*	
C14	0.1411 (3)	0.9312 (7)	0.2714 (2)	0.0380 (10)	
H14	0.165837	0.938978	0.225560	0.046*	
C15	0.1615 (3)	0.7687 (6)	0.3217 (2)	0.0290 (9)	
C16	0.2621 (5)	0.6219 (9)	0.2449 (3)	0.078 (2)	
H16A	0.209173	0.634567	0.191795	0.118*	
H16B	0.301214	0.505956	0.244719	0.118*	
H16C	0.306739	0.733112	0.253872	0.118*	
N1	0.3202 (2)	0.2815 (5)	0.46627 (18)	0.0309 (7)	
N2	0.1476 (2)	0.5848 (5)	0.43945 (19)	0.0302 (7)	
HN2	0.173630	0.491051	0.418615	0.036*	
O1	0.4653 (2)	0.3005 (4)	0.57658 (16)	0.0363 (7)	
O2	0.0961 (3)	0.6653 (6)	0.55175 (19)	0.0687 (11)	
O3	0.2163 (2)	0.6103 (4)	0.31065 (17)	0.0467 (8)	
S1	0.29895 (9)	0.35394 (17)	0.62002 (6)	0.0420 (3)	

### Atomic displacement parameters (Å^2^)

**Table:**

	*U*^11^	*U*^22^	*U*^33^	*U*^12^	*U*^13^	*U*^23^
C1	0.036 (2)	0.0217 (18)	0.032 (2)	0.0038 (16)	0.0093 (17)	0.0053 (16)
C2	0.047 (3)	0.045 (3)	0.039 (2)	0.002 (2)	0.013 (2)	0.000 (2)
C3	0.059 (3)	0.046 (3)	0.058 (3)	0.006 (2)	0.033 (3)	0.001 (2)
C4	0.044 (3)	0.036 (2)	0.085 (4)	0.005 (2)	0.035 (3)	0.009 (2)
C5	0.032 (2)	0.035 (2)	0.066 (3)	−0.0031 (19)	0.007 (2)	0.009 (2)
C6	0.038 (2)	0.0217 (19)	0.039 (2)	−0.0024 (17)	0.0112 (18)	0.0046 (17)
C7	0.036 (2)	0.024 (2)	0.029 (2)	0.0016 (16)	0.0067 (17)	0.0061 (16)
C8	0.041 (2)	0.052 (3)	0.034 (2)	−0.009 (2)	0.0208 (19)	0.000 (2)
C9	0.036 (2)	0.053 (3)	0.026 (2)	−0.001 (2)	0.0156 (18)	−0.0053 (19)
C10	0.0190 (18)	0.040 (2)	0.0195 (18)	−0.0011 (16)	0.0007 (15)	−0.0058 (16)
C11	0.027 (2)	0.050 (3)	0.030 (2)	0.0069 (19)	0.0060 (17)	−0.0084 (19)
C12	0.035 (2)	0.049 (3)	0.040 (3)	0.017 (2)	0.002 (2)	−0.010 (2)
C13	0.043 (3)	0.044 (3)	0.038 (2)	0.012 (2)	0.000 (2)	0.005 (2)
C14	0.034 (2)	0.055 (3)	0.022 (2)	0.004 (2)	0.0055 (17)	0.0048 (19)
C15	0.0247 (19)	0.040 (2)	0.0224 (18)	0.0045 (17)	0.0067 (15)	−0.0009 (17)
C16	0.117 (5)	0.081 (4)	0.066 (4)	0.054 (4)	0.071 (4)	0.027 (3)
N1	0.0325 (18)	0.0342 (18)	0.0236 (17)	0.0008 (14)	0.0055 (14)	−0.0010 (14)
N2	0.0334 (18)	0.0369 (18)	0.0261 (16)	0.0024 (14)	0.0177 (14)	−0.0058 (14)
O1	0.0348 (16)	0.0358 (15)	0.0311 (15)	−0.0026 (13)	−0.0002 (12)	0.0042 (12)
O2	0.101 (3)	0.082 (3)	0.0401 (19)	0.035 (2)	0.046 (2)	0.0056 (18)
O3	0.062 (2)	0.0517 (19)	0.0380 (17)	0.0240 (16)	0.0333 (15)	0.0094 (14)
S1	0.0516 (7)	0.0517 (7)	0.0226 (5)	0.0004 (6)	0.0114 (5)	0.0050 (5)

### Geometric parameters (Å, º)

**Table:**

C1—C6	1.375 (6)	C9—O2	1.211 (5)
C1—C2	1.389 (5)	C9—N2	1.348 (5)
C1—N1	1.401 (5)	C10—C11	1.388 (5)
C2—C3	1.377 (6)	C10—C15	1.406 (5)
C2—H2	0.9300	C10—N2	1.408 (5)
C3—C4	1.382 (7)	C11—C12	1.381 (6)
C3—H3	0.9300	C11—H11	0.9300
C4—C5	1.379 (7)	C12—C13	1.370 (6)
C4—H4	0.9300	C12—H12	0.9300
C5—C6	1.370 (6)	C13—C14	1.377 (6)
C5—H5	0.9300	C13—H13	0.9300
C6—O1	1.388 (5)	C14—C15	1.374 (6)
C7—N1	1.278 (5)	C14—H14	0.9300
C7—O1	1.362 (5)	C15—O3	1.366 (4)
C7—S1	1.740 (4)	C16—O3	1.426 (5)
C8—C9	1.514 (6)	C16—H16A	0.9600
C8—S1	1.812 (4)	C16—H16B	0.9600
C8—H8A	0.9700	C16—H16C	0.9600
C8—H8B	0.9700	N2—HN2	0.8600
			
C6—C1—C2	119.4 (4)	C11—C10—N2	124.6 (3)
C6—C1—N1	109.4 (3)	C15—C10—N2	116.7 (3)
C2—C1—N1	131.2 (4)	C12—C11—C10	120.4 (4)
C3—C2—C1	117.2 (4)	C12—C11—H11	119.8
C3—C2—H2	121.4	C10—C11—H11	119.8
C1—C2—H2	121.4	C13—C12—C11	120.4 (4)
C2—C3—C4	121.8 (4)	C13—C12—H12	119.8
C2—C3—H3	119.1	C11—C12—H12	119.8
C4—C3—H3	119.1	C12—C13—C14	120.0 (4)
C5—C4—C3	121.8 (4)	C12—C13—H13	120.0
C5—C4—H4	119.1	C14—C13—H13	120.0
C3—C4—H4	119.1	C15—C14—C13	120.6 (4)
C6—C5—C4	115.3 (4)	C15—C14—H14	119.7
C6—C5—H5	122.3	C13—C14—H14	119.7
C4—C5—H5	122.3	O3—C15—C14	125.4 (3)
C5—C6—C1	124.5 (4)	O3—C15—C10	114.6 (3)
C5—C6—O1	128.0 (4)	C14—C15—C10	120.0 (4)
C1—C6—O1	107.4 (3)	O3—C16—H16A	109.5
N1—C7—O1	117.4 (3)	O3—C16—H16B	109.5
N1—C7—S1	128.1 (3)	H16A—C16—H16B	109.5
O1—C7—S1	114.5 (3)	O3—C16—H16C	109.5
C9—C8—S1	111.7 (3)	H16A—C16—H16C	109.5
C9—C8—H8A	109.3	H16B—C16—H16C	109.5
S1—C8—H8A	109.3	C7—N1—C1	103.1 (3)
C9—C8—H8B	109.3	C9—N2—C10	127.4 (3)
S1—C8—H8B	109.3	C9—N2—HN2	116.3
H8A—C8—H8B	107.9	C10—N2—HN2	116.3
O2—C9—N2	124.7 (4)	C7—O1—C6	102.7 (3)
O2—C9—C8	120.1 (4)	C15—O3—C16	116.7 (3)
N2—C9—C8	115.2 (3)	C7—S1—C8	98.82 (18)
C11—C10—C15	118.7 (4)		
			
C6—C1—C2—C3	−0.6 (6)	N2—C10—C15—O3	−1.0 (5)
N1—C1—C2—C3	−178.6 (4)	C11—C10—C15—C14	0.2 (5)
C1—C2—C3—C4	0.4 (7)	N2—C10—C15—C14	179.1 (3)
C2—C3—C4—C5	0.1 (7)	O1—C7—N1—C1	1.2 (4)
C3—C4—C5—C6	−0.5 (6)	S1—C7—N1—C1	−177.6 (3)
C4—C5—C6—C1	0.3 (6)	C6—C1—N1—C7	−0.9 (4)
C4—C5—C6—O1	178.3 (4)	C2—C1—N1—C7	177.2 (4)
C2—C1—C6—C5	0.3 (6)	O2—C9—N2—C10	0.9 (7)
N1—C1—C6—C5	178.7 (4)	C8—C9—N2—C10	−178.8 (3)
C2—C1—C6—O1	−178.1 (3)	C11—C10—N2—C9	−10.6 (6)
N1—C1—C6—O1	0.3 (4)	C15—C10—N2—C9	170.5 (4)
S1—C8—C9—O2	−88.1 (5)	N1—C7—O1—C6	−1.1 (4)
S1—C8—C9—N2	91.6 (4)	S1—C7—O1—C6	177.9 (2)
C15—C10—C11—C12	0.5 (6)	C5—C6—O1—C7	−177.9 (4)
N2—C10—C11—C12	−178.3 (4)	C1—C6—O1—C7	0.4 (4)
C10—C11—C12—C13	−1.0 (6)	C14—C15—O3—C16	4.8 (6)
C11—C12—C13—C14	0.7 (7)	C10—C15—O3—C16	−175.1 (4)
C12—C13—C14—C15	0.1 (6)	N1—C7—S1—C8	−0.8 (4)
C13—C14—C15—O3	179.7 (4)	O1—C7—S1—C8	−179.7 (3)
C13—C14—C15—C10	−0.5 (6)	C9—C8—S1—C7	−87.7 (3)
C11—C10—C15—O3	−180.0 (3)		

### Hydrogen-bond geometry (Å, º)

*Cg*3 is the centroid of the C10–C15 benzene ring of the methoxy phenyl group.

**Table:**

*D*—H···*A*	*D*—H	H···*A*	*D*···*A*	*D*—H···*A*
N2—H*N*2···O3	0.86	2.22	2.608 (4)	107
N2—H*N*2···N1	0.86	2.39	3.075 (4)	136
C8—H8*A*···N1	0.97	2.48	2.914 (5)	107
C11—H11···O2	0.93	2.28	2.869 (5)	121
C12—H12···O2^i^	0.93	2.52	3.378 (6)	153
C13—H13···*Cg*3^ii^	0.93	2.89	3.634 (5)	138

Symmetry codes: (i) −*x*, −*y*+2, −*z*+1; (ii) −*x*, *y*+1/2, −*z*+1/2.
